# A Comparison of the Efficacy, Adverse Effects, and Patient Compliance of the Sena-Graph®Syrup and Castor Oil Regimens for Bowel Preparation 

**Published:** 2010

**Authors:** Karim Ghazikhanlou Sani, Mahmood-Reza Jafari, Safar Shams

**Affiliations:** a *Department of Radiology, Hamadan University of Medical Sciences, Hamadan, Iran. *; b *Department of Laboratory Sciences, Hamadan University of Medical Sciences, Hamadan, Iran.*

**Keywords:** Bowel evacuants, Castor oil, Intravenous urography, Sena-Graph, Patient compliance

## Abstract

Sena-Graph syrup has recently been formulated by an Iranian pharmaceutical company for being used in bowel evacuation before radiography, colonoscopy and surgery. This study compares the efficacy, adverse effects and patient compliance of two bowel preparation regimens with castor oil and Sena-Graph syrup in of outpatients for Intravenous Urography (IVU).

One hundred and fourteen consecutive outpatients were randomized to receive either the standard bowel preparation with 60 mL of castor oil or the test method with 60 mL of Sena-Graph syrup before IVU examination. Demographic data of patients and their prior bowel preparation experience were collected before the examination. Two radiologists, blinded to the method of bowel preparation, reviewed the radiographs and graded the bowel preparation. The compliance and acceptability of both regimens were assessed by using structured questionnaires filled by the patients.

The Numbers, ages, weights and gender distribution of patients and their prior bowel preparation experience in the two groups did not differ significantly. The cleanliness scores for the castor oil and Sena-Graph group were 3.97 ± 0.971 and 4.87 ± 0.917, respectively. The results indicated that Sena-Graph syrup causes a better bowel cleansing compared castor oil. Adverse effects in Sena-Graph groups were significantly lower than the castor oil group. Acceptability of the regimen in patients who used Sena-Graph was higher than the other group.

The Sena-Graph regimen is significantly more effective and better tolerated than of Castor oil regimen in bowel cleansing. The incidence and severity of the adverse effects from Castor oil was higher than Sena-Graph.

## Introduction

The use of laxatives has been present from the early 19th century (1). Today, the most common indications for the use of laxatives are their therapeutic, as well as diagnostic uses (1, 2). Bowel preparation has long been considered necessary to improve the diagnostic quality of radiological examinations of abdominal region such as Intravenous Urography (IVU) and colonoscopy (3-6). In addition, an inadequate colon preparation often makes repeated examination necessary, which results in inconvenience and high cost for the patients (7, 8). For example, approximately one out of five incomplete colonoscopies is the result of inadequate bowel preparation (9). Almost since their introduction, laxatives have been used for bowel cleansing and there has been controversy over the methods utilized to prepare the colon for the examination (9, 10). Especially, in the past two decades, various bowel cleansing methods have been proposed (11). An ideal colon preparation must provide a safe and rapid cleansing acceptable to the patients, with little or no discomfort and side effects. However, most of the presently available methods do not have these criteria (5, 11). 

Castor oil is commonly used as a preparation means for radiological and colonoscopy examinations, but its aftertaste and oily texture are intolerable by most patients (7). Also, castor oil is more likely to cause adverse effects such as abdominal cramping, vomiting, nausea, abdominal fullness, fainting and insomnia (7, 10). Since, in 35% of the cases, patient discomfort is a major factor of failed colon preparations (9, 12), replacement of a well tolerated method for bowel cleansing seems to be necessary (5).

Recently Sena-Graph syrup has been formulated by an Iranian pharmaceutical company for being used in bowel evacuation before radiography, colonoscopy and surgery. Sena-Graph is a 60 mL watery texture syrup (consisting *Cassia angustifolia*, sugar, sorbitol, sodium citrate, citric acid, propylene glycol, propylene paraben, methyl paraben, lemon essence, rosewater and water), 100 mL of which contains 200 mg sennosides A and B (13). Senna extract is a cathartic obtained from *Cassia acutifolia *and *Cassia augustifolia *(10). 

The purpose of this study was to compare the bowel cleansing efficacy, adverse effects and patient compliance of the two laxatives, castor oil and Sena-Graph syrup, in the routine bowel preparation of outpatients for Intravenous Urography (IVU). 

## Experimental

One hundred and fourteen consecutive outpatients who were scheduled for IVU examination were randomized to enroll into the study (57 patients in Sena-Graph group and 57 in castor oil group). As there was not any similar study, an exact estimation of the sample size was not possible. Therefore, because of the time limitations a six months period was considered as adequate for the study. All of the patients who referred to the Ekbatan Hospital (Hamadan University of Medical Sciences) for IVU examination during the 6 months period from April 2008 to September 2008, and who completed the informed consent form, were registered in the study. Single random sampling method was used in this study and the patients received either the castor oil or the Sena-Graph regimen. According to recommendations of producers, pregnant and nursing women, children less than 6 years, and patients with GI obstruction or GI inflammatory disorders were excluded from the study (13). A written informed consent form that was approved by the Ethics Committee of Hamadan University of Medical Sciences, was designed in Persian and signed by all of the participants. 

Eligible patients were randomized to receive either the standard bowel preparation by 60 mL of castor oil or the test preparation by 60 mL of Sena-Graph syrup. Both groups were instructed to start the preparation around 4:00 p.m. the day before the IVU which was scheduled after 8:00 a.m. 

Demographic data such as the age, weight, gender, prior bowel preparation experience, and medical history were recorded for all of the participants. After obtaining a plain image of the abdomen as the first step of IVU examination, two radiologists who were blinded to the bowel preparation regimens reviewed and graded the radiographs separately and independently. To evaluate the degree of fecal residue on the plain abdominal images, a grading system was made. When no residual fecal material was seen in a specified area of the film (region between xiphoid, symphysis pubis and iliac crests), the score was 3; when residual materials were seen in less than one-third of the specified area, the score was 2; and if the residue all the material were seen in less than two thirds, and more than one-third of the specified film area, the score was recorded as 1; and finally the score was reported as 0, if there was residue in more than two-thirds of the area (4). 

A self-administered structured questionnaire was completed by each patient to assess the adverse effects. The evaluated adverse effects in this study included nausea, vomiting, abdominal pain, abdominal fullness, thirst, fainting, diarrhea, anal irritation, and insomnia. Severity of these effects was graded as none, mild, moderate and severe according to the patient’s claims. The illiterate patients were interviewed, and the questions and possible answers were read for them in a loud voice. 

Patient compliance, palatability and acceptability of the laxatives were evaluated by using the same questionnaire. Patients’ compliance was considered positive if they had been able to complete the bowel preparation regimen. The taste and palatability were graded as desirable and undesirable. Patient acceptance was positive if they would like to utilize the same regimen again. 

The design of the questionnaire for evaluating the adverse effects and patient compliance was adapted from the works of Guo et al. (14), Hwang et al. (11), and Chen et al. (7). The reliability of the questionnaires was approved by statistical analysis (Chronbach’s Alpha = 0.704). The bowel preparation scoring system was based on the work of Yang et al. (4). Also two pathologists and one radiologist, who were experts at research, evaluated the questionnaires for content validity.

The statistical analysis was performed using the SPSS software, version 13. Demographic data were expressed as mean ± SD. The Student’s t-test was used to compare the bowel cleansing scores between the two groups. The Pearson chi square and Likelihood Ratio tests were used to compare the incidence and severity of adverse effects between the two groups. Patient compliance, acceptability, and palatability of the two laxatives were analyzed by using Fishers exact test. P < 0.05 was defined as being statistically significant.

## Results and Discussion

In this study, 57 outpatients were given castor oil as the laxative for bowel preparation, and 57 were given Sena-Graph syrup. The patients demographic data with regard to age, gender and weight are summarized in Table 1.

**Table 1:** The number, age and weight distribution of participants in each group

	Sena-Graph (n = 57)		Castor oil (n = 57)		P-value*
	Men (n = 20) (Mean ± SD)	Women (n = 37) (Mean ± SD)	Men (n = 21) (Mean ± SD)	Women (n = 36) (Mean ± SD)	
Age	14.13 ± 42.65	18.43 ± 49.50	16.22 ± 44.14	12.59 ± 41.29	0.499
Weight	12.95 ± 76.84	10.62 ± 61.85	12.93 ± 72.89	12.34 ± 65.84	0.578

* One-way ANOVA

 No significant difference in any of these variables was observed between the two groups, although slight difference was observed between the ages of the two groups. Twenty five participants in castor oil group and 22 participants in Sena group had prior bowel preparation experience. Statistical analysis did not show any significant difference in this variable as well (p-value = 0.572). 

The patients who take Sena-Graph for bowel preparation showed significantly better bowel cleansing compared to the other group. The mean bowel cleanliness score for the Sena-Graph group was 4.87 ± 0.917, compared to 3.97 ± 0.971 for the castor oil group. Bowel cleansing scores are shown in Table 2. Note that two radiologists scored the bowel cleansing on a 0-3 scale, so the sum of the two scores was in the range of 0 to 6. 

**Table 2:** Comparison of the bowel cleansing scores in each bowel preparation regimens

	Sena-Graph number of patients (%)	Castor oil number of patients (%)
≤2	4 (7.01)	14 (24.56)
3-4	29 (50.88)	30 (52.63)
5-6	24 (42.11)	13 (22.81)

* Student’s t test

(p-value= 0.000)

The analysis the bowel cleansing scores indicated that there is a statistically significant difference between two groups (p-value =0.000). 

The incidence and severity of the adverse effects (including nausea, vomiting, abdominal pain, abdominal fullness, thirst, fainting, diarrhea, anal irritation and insomnia) in the castor oil and Sena-Graph groups are summarized in Table 3. Pearson chi square and likelihood ratio tests were used for determination of the statistical significant differences. 

**Table 3:** The occurrence and severity of the Sena-Graph anticipated adverse effects in each group.

	Sena-Graph				Castor oil				P-Value**
	Mild	Moderate	Sever Moderate	Occurrence*(%)	Mild	Moderate	Sever	Occurrence* (%)	
Nausea^1,2 ^	18	1	1	20 (35.0)	13	19	0	32 (56.1)	0.001
Vomiting^1^	14	5	0	19 (33.3)	14	17	0	31 (54.4)	0.012
0.012Abdominal pain^1^	19	17	0	36 (63.2)	13	23	0	36 (63.2)	0.421
Thirst^1^	16	2	0	18 (31.6)	20	12	0	32 (56.1)	0.020
Fainting^1 ^	20	2	0	22 (38.6)	15	9	0	24 (42.1)	0.139
0.139Abdominal fullness^2^	18	6	0	24 (42.1)	13	26	6	39 (68.4)	0.001
0.001Anal irritation^1^	28	25	0	53 (92.9)	16	14	0	30 (52.6)	0.001
Diarrhea^1,2 ^	3	37	17	57 (100)	4	22	26	52 (91.2)	0.003
Insomnia^1^	6	1	0	7 (12.3)	20	9	0	29 (50.8)	0.001

* The occurrence rate was calculated by “frequency of occurrence/total number of subjects.

** Pearson chi square_1_ and Likelihood ratio_2_ tests were used for Statistical analysis.

**Figure 1:**
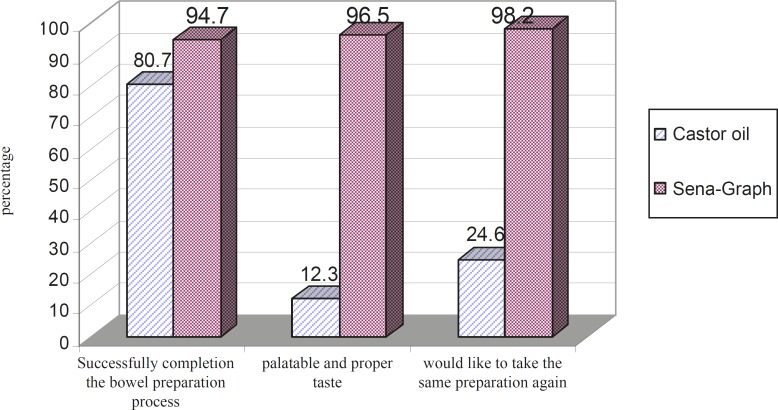
The comparison of the tolerance, palatability and acceptability of the two bowel preparation regimens

The incidence and severity of some of the adverse effects (i.e. vomiting, nausea, thirst, abdominal fullness, fainting and insomnia) was significantly higher in castor oil group Table 3. However, the incidence and severity of anal irritation was higher in Sena-Graph group. Although the incidence of diarrhea was higher in Sena-Graph group but its severity was higher in castor oil group. A comparison of tolerance, palatability and acceptability of Sena-Graph and castor oil regimens is shown in Figure 1.

Significant differences were observed in compliance, palatability and acceptability of Sena-Graph and castor oil regimens. Evaluation of the taste of the two alternatives showed that most of the patients disliked castor oil and did not like to have this preparation again, while the majority of the patients enjoyed the taste of Sena-Graph. Most of the patients in Sena-Graph group had completed the bowel preparation process, whereas 11 patients in castor oil group (19.3%) could not swallow the castor oil completely. When the patients were asked whether they would take the same preparation in the future, 56 in the Sena-Graph group (98.2%) replied “yes”, compared to only 14 in the castor oil group (24.6%). Statistical analysis by Fisher’s exact test revealed a significant relationship between the patients’ acceptability and the type of bowel preparation regimens (p-value = 0.43). 

Most of the published studies have reported that the castor oil regimen is an improper regimen for bowel preparation and causes adverse effects (4, 7, 9, 12, 14-16). Huei-Chen Yang *et al. *(4) showed that excellent or good cleansing was achieved in 60% of patients in castor oil group while 40% of the patients had poor preparation. Also, some previous studies have reported that good bowel cleansing occurs in 54–62% of the castor oil consumers (15, 16). The results of the present study demonstrated that the Sena-Graph regimen was better than castor oil regimen for bowel preparation. Our findings indicated that about 65% of the patients in castor oil group had good bowel preparation (score ≥3). This value was 81% in Sena-Graph group (score≥3). These differences between our results and those of the other studies may be related to the different grading system. 

The reports of the adverse effects in those patients receiving castor oil regimen for bowel preparation are in good agreement with results reported by Chun-Chia Chen et al (7). The mean incidence of the adverse effects (e.g. nausea, vomiting, abdominal pain, abdominal fullness and fainting) in their study was 22% (range 18–29%) compared to 28% (range 24–39%) in this study. Although the occurrence of some of the adverse effects (such as, vomiting, nausea, thirst, abdominal fullness, fainting and insomnia) was higher in castor oil regimen, the incidence and severity of anal irritation was higher in Sena- Graph regiment. However, the results showed that the adverse effects occurred more often in patients undergoing castor oil regimen. 

To the best of our knowledge, there was not any similar study about efficacy and acceptability of Sena-Graph in Iran. Although there were some similar investigations about senna extract in other countries, due to some differences between the Sena-Graph syrup and the pure senna extract, their results could not apply to the Sena-Graph syrup. 

In conclusion, there are significant differences in efficacy, adverse effects and tolerability of Sena-Graph and castor oil regimens. Sena- Graph syrup was clearly and proved to be safe and effective in bowel cleansing for radiological examinations. 
